# The Long Non-coding RNA AC148477.2 Is a Novel Therapeutic Target Associated With Vascular Smooth Muscle Cells Proliferation of Femoral Atherosclerosis

**DOI:** 10.3389/fcvm.2022.954283

**Published:** 2022-07-06

**Authors:** Kangjie Wang, Yanchen Ye, Lin Huang, Ridong Wu, Rongzhou He, Chen Yao, Shenming Wang

**Affiliations:** ^1^Division of Vascular Surgery, The First Affiliated Hospital, Sun Yat-sen University, Guangzhou, China; ^2^National-Guangdong Joint Engineering Laboratory for Diagnosis and Treatment of Vascular Disease, First Affiliated Hospital, Sun Yat-sen University, Guangzhou, China

**Keywords:** arteriosclerosis obliterans, vascular smooth muscle cells, long non-coding RNA, superficial femoral artery, femoral atherosclerosis

## Abstract

Arteriosclerosis obliterans (ASO) is a limb manifestation of large vessel atherosclerosis. Phenotype switching of vascular smooth muscle cells (VSMCs) occurs in the course of the pathological process. The underlying mechanism of SMCs proliferation remains unclear. Several studies have demonstrated that the dysregulation of long non-coding RNA (lncRNAs) plays a pivotal part in the progression of ASO by exacerbating the proliferation of VSMCs. Based on the endogenous competitive RNA (ceRNA) hypothesis, the mechanism of lncRNAs involved in the pathology of VSMCs was exposed, while the entire map of the regulatory network remains to be elucidated. In the current study, genes and the lncRNAs modules that are relevant to the clinical trait were confirmed through weighted gene co-expression network analysis (WGCNA). In this study, we comprehensively constructed a specific lncRNAs-mediated ceRNA and RBP network. The three lncRNAs, *HMGA1P4, C5orf66*, and *AC148477.2*, influenced the proliferation of VSMCs and were found to be associated with the immune landscape, thus they were ultimately screened out. Further verification revealed that *AC147488.2* was significantly down-regulated in both ASO arteries and all stages of proliferative VSMCs, which implied that *AC147488.2* might have a significant impact on ASO. This finding would improve our understanding of the epigenetic regulation of ASO and unravel novel diagnostic and therapeutic targets.

## Introduction

Arteriosclerosis obliterans (ASO) is a clinical manifestation of systemic atherosclerosis that primarily results in the occlusion of lower limb arteries with different degrees (1). ASO is the third leading cause of atherosclerotic cardiovascular morbidity. It has raised a great public health concern owing to its increasing incidence in most developed countries especially in the elderly population (2). The most common artery that is affected in the lower limb is the superficial femoral artery (SFA) (3). However, the essential molecular mechanism of ASO needs to be elucidated. The complications of ASO include lipid deposition, endothelial damage, inflammatory response, cell proliferation, and metabolic disorders (4). Different cell types are involved in the formation of femoral atherosclerosis (5–8).

Vascular smooth muscle cells (VSMCs), one of the major classes of differentiated cells of the vascular wall, primarily reside in the tunica media. The VSMCs are generally in a quiescent state and control the vascular tone and blood distribution under normal situations. When stimulated by inflammatory cytokines or injuries, VSMCs will be dynamically transformed into the proliferative phenotype with remarkable plasticity and a reduction of the contractility-associated markers (e.g., MYH11, ACTA2) (9). In the past decade, lineage-tracking strategies have indicated that proliferative VSMCs increasingly migrate into the tunica intima and synthesize extracellular matrix components (10). The proliferation of VSMCs eventually results in the formation of neointima, which governs the process of atherosclerosis lesion progression. Strategies that restrict the proliferation of VSMCs have been studied with the aim of treating atherosclerosis.

Long non-coding RNAs (lncRNAs) are RNA molecules that are longer than 200 nucleotides in length but they lack the protein-coding potential (11). LncRNAs directly regulate proteins at the epigenetic, transcriptional, and post-transcriptional levels through diverse mechanisms (12, 13). Recently reported evidence has shown that lncRNAs play an essential role in the pathophysiological progression of atherosclerosis (14, 15). The competitive endogenous RNA hypothesis, which was initially proposed by Salmena et al., suggests that lncRNAs act as molecular sponges and bind to the miRNAs response elements to regulate downstream gene expression (16). In recent years, lncRNAs have been demonstrated in the functional regulation of VSMCs proliferation. For instance, *RNCR3* can regulate VSMCs proliferation by regulating the crosstalk between Krüppel-like factor 2 and *miR-185-5p* (17). Moreover, the lncRNA *SMILR* was reported to modulate VSMCs proliferation by targeting the *miR-10b-3p*/KLF5 axis (18). The comprehensive understanding of the lncRNAs-mediated ceRNA network, especially in proliferative VSMCs of femoral atherosclerosis, has not been well elucidated.

In this study, a specific ceRNA network and an RBP network that included three lncRNAs were established with the aim of highlighting the role of proliferative VSMCs in femoral atherosclerosis.

## Materials and Methods

### Gene Expression Omnibus Dataset Acquisition and Processing

The dene expression profile of femoral atherosclerosis was collected from the online source database of GEO^1^. After removing an outlier sample, GSE100927, including 37 femoral artery samples, was selected for analysis. Another microarray dataset (GSE77279) was also included. It analyzed the lncRNAs and mRNAs profiles of proliferative aortic smooth muscle cells (HASMCs). All RNA probes were re-annotated by the R package “AnnoProbe.”

### Weighted Gene Co-expression Network Analysis

The construction of the weighted gene co-expression network was performed by R the package “WGCNA” (19) with default parameters. In brief, the gene expression matrixes included genes that were in the top 5,000 expressed before the median absolute deviation was imported for analysis. The soft threshold power was set as 4 and 5 (scale-free *R*^2^ = 0.85) for the matrix of genes and lncRNAs. Next, the topological overlap matrix (TOM) was employed to measure the similarity between genes. Afterward, genes with high correlation were hierarchically clustered into the same module and visualized in a dendrogram based on the dissimilarity (1-TOM). An outlier sample was found and removed. The module eigengene (ME) representing each module was calculated to estimate the module-trait associations with atherosclerosis. The expressions of module genes were visualized in a heatmap by the R package “pheatmap.” Genes with a threshold of module membership |MM| > 0.8 and gene significance |GS|>0.2 were considered the key module genes for subsequent analysis.

### Identification of Differentially Expressed lncRNAs and Genes

The gene expression matrices of the proliferative and quiescent VSMCs were compared by utilizing the R package “limma” (20). The genes and lncRNAs were classified as differentially expressed genes (DEGs) and lncRNAs (DElncRNAs) based on the threshold value |*logFC*| ≥ 1 and an *adjusted. p* < 0.05. Visualization of the expression of DEGs and DElncRNAs was conducted by the R package “ggplot2.”

### Functional Annotation of Module Genes

Gene ontology (GO) of biological processes and Kyoto Encyclopedia of Genes and Genomes (KEGG) pathway enrichment analysis for DEGs were performed by the R package “ClusterProfiler” (21). The top 10 significantly different GO terms and signal pathways were visualized in scatter plots with the threshold *p* < 0.05 and *adjusted. p* < 0.05. Genes of the interesting modules analyzed by WGCNA were also applied for GO and KEGG pathways enrichment. The gene set enrichment analysis (GSEA) of related pathways in GSE100927 was performed by the R package “ClusterProfiler”. Gene list ranked by *logFC* was the input for analysis. The significant pathways were shown with the threshold *adjusted. p* < 0.05.

### Construction of the Specific Protein-Protein Interaction Network of Proliferative Vascular Smooth Muscle Cells in Femoral Atherosclerosis

To further identify the pivotal genes that probably contributed to VSMCs proliferation in module genes, the common genes between module genes and DEGs, defined as the specific proliferative VSMCs genes (sp-SMGs), were detected and visualized by the R package “VennDiagram”. Next, the online database STRING (22) was applied for the prediction of protein–protein interaction (PPI) networks, which was visualized utilizing Cytoscape software (version 3.8.0) (23). The hub genes of the PPI network were calculated by five algorithms of the cytoHubba plug-in: MCC, MNC, EPC, Degree, and Closeness.

### Construction of the Specific Competitive RNA and RNA Binding Proteins Network of Proliferative Vascular Smooth Muscle Cells in Femoral Atherosclerosis

The specific proliferative VSMCs genes (sp-SMGs) and lncRNAs (sp-SMlncRNAs) were selected to establish the ceRNA network. The lncRNA–miRNA interaction pairs were predicted by the ENCORI database (24). The miRNA–mRNA interaction pairs were screened by miRanda (25), Targetscan (26), and ENCORI databases. Thereafter, the ceRNA network, which was constituted by lncRNAs, miRNAs, and mRNAs was constructed and visualized by the Cytoscape software. For the RBP network part, potential lncRNA-RBP and mRNA RBP pairs that were validated by the experiment were retained from the ENCORI database. The specific RBP network was structured by the Cytoscape software.

### Tissue Acquisition and Quantitative Real-Time Polymerase Chain Reaction

The acquisition procedure was approved by the IEC for Clinical Research and Animal Trials of the First Affiliated Hospital of Sun Yat-sen University (approval no. [2021]668). In total, 12 ASO arterial samples were obtained from the patients who were diagnosed with arteriosclerosis obliterans and suffered from critical lower limb ischemia. Superficial femoral arteries were separated after amputation. For the normal artery acquisition, 12 healthy donors without a history of ASO disease or arteriostenosis were chosen. After isolation of SFA, only those arteries with the normal vascular structure were retained for follow-up experiments. Primary human artery smooth muscle cells (HASMCs) were obtained from the femoral artery of a healthy organ donor. To verify the expression level of three lncRNAs in an *in vitro* model, HASMCs were made quiescent for 24 h of culture in serum-free DMEM medium. After that, HASMCs were treated with or without PDGF-BB (PeproTech Inc.) at a concentration of 20 ng/ml for 24 h to simulate early lesions. Stimulation with ox-LDL (100 μg/ml, Yiyuan Biotech. Co., Ltd.) and TNF-α (20 ng/ml, Abbkine Scientific Co., Ltd.) for 24 h was another approach to simulate the conditions of advanced diseases. Total RNA was extracted from arterial specimens or cells using TRIzol reagent (Invitrogen, Thermo Fisher Scientific, Co., Ltd.). RNA was reverse transcribed into cDNA using an Evo M-MLV Mix Kit (Accurate Biology, AG11728, Hunan, China) according to the manufacturer’s instructions. The expressions of the lncRNAs were determined with the SYBR Green Premix Pro Taq HS qPCR Kit (Accurate Biology, AG11701, Hunan, China). All experiments were performed in triplicates.

### Immune Filtration Analysis and Correlation Between Long Non-coding RNA and Immune Cells

To gain insight into the immune infiltration landscape of femoral atherosclerosis, two different methods were chosen. The single-sample gene set enrichment analysis (ssGSEA) was performed with the comprehensive evaluation of the expression levels of immune cell-specific marker genes that were retrieved from the published article (27). The CIBERORT website analytical tool was employed to assess the immune landscape based on the LM22 immune signature gene set (28). The association of the identified lncRNAs with immune cells was computed through Pearson’s correlation analysis.

### Statistical Analyses

The data were analyzed with SPSS 20.0, and the significance of the difference was determined using the two-tailed Student’s *t*-test or the Mann–Whitney *U*-test. The odd ratio of three lncRNAs was calculated by univariate logistics regression and visualized in the forest plot. To examine the specific lncRNAs performance, the R package “pROC” was employed to compare the areas under the receiver operating characteristic (ROC) curves (AUC).

## Results

### Construction of Weighted Co-expression Networks

**FIGURE 1:**
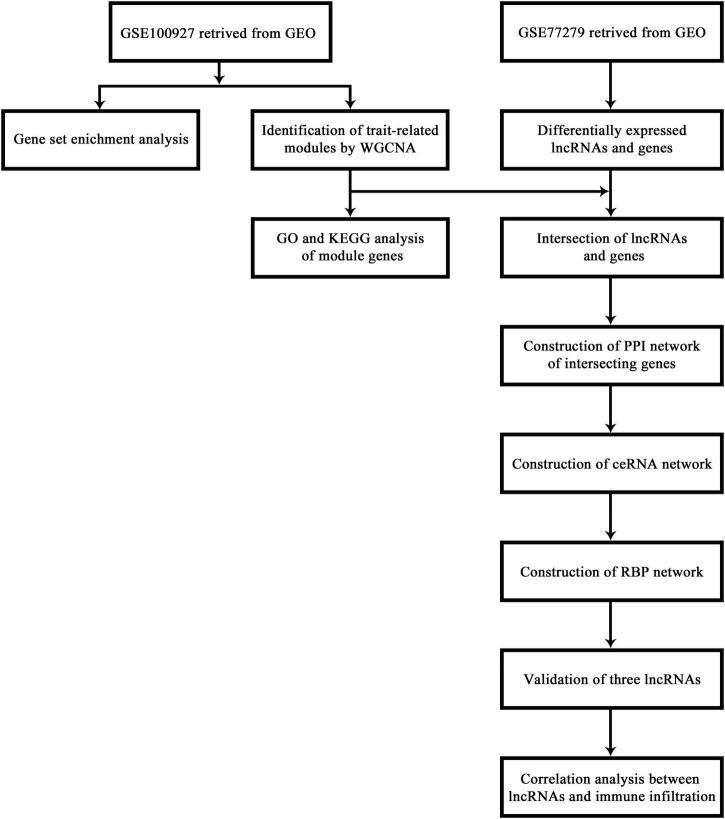
The flow chart of the procedures carried out in this study.

**FIGURE 2:**
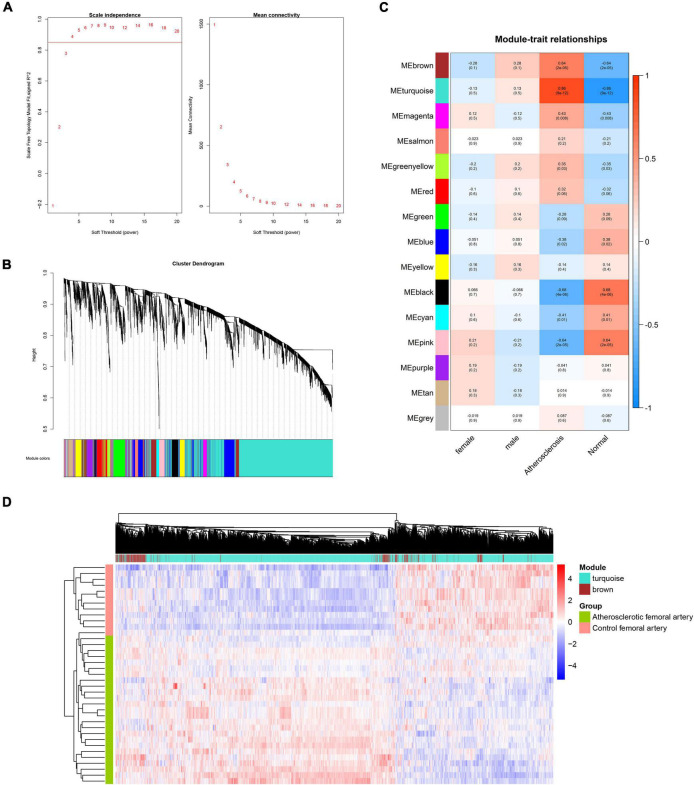
WGCNA analysis of genes in femoral atherosclerosis. **(A)** The chosen soft threshold power of genes matrix. **(B)** The cluster dendrogram of gene module. **(C)** The correlation heatmap of module-trait relationships. The first-row number in each block indicates the correlation coefficient. The number in parentheses represents the *p*-value. **(D)** Heatmap displays the expression of genes in the screened modules between two groups.

The flow chart of this research study is shown in Figure 1. To further determine which genes were highly associated with femoral atherosclerosis, WGCNA was performed. One outlier was removed during the hierarchical clustering tree construction (Supplementary Figure 1). For genes, β = 4 was selected as the soft threshold power to gratify the construction of a scale-free network (Figure 2A). Ultimately, 15 gene modules were identified and visualized in a cluster dendrogram (Figure 2B). Gray modules included genes that were eliminated from any module. The correlation between gene modules and clinical traits was analyzed and displayed in a correlation heatmap (Figure 2C). The brown and turquoise modules, comprised of 371 and 2,488 genes, respectively, were considered key modules for subsequent analysis as the two modules were positively associated with atherosclerosis (*r* > 0.5, *p* < 0.05). The expression of two module genes is shown in the heatmap, which indicates that those genes may be differentially expressed between atherosclerotic femoral arteries and control femoral arteries (Figure 2D). The enrichment results suggested that the brown module genes mainly participated in the cellular matrix organization and cell adhesion-relevant signaling pathways. The turquoise module genes are primarily involved in the immune cell activation and chemokine signaling pathways.

**FIGURE 3:**
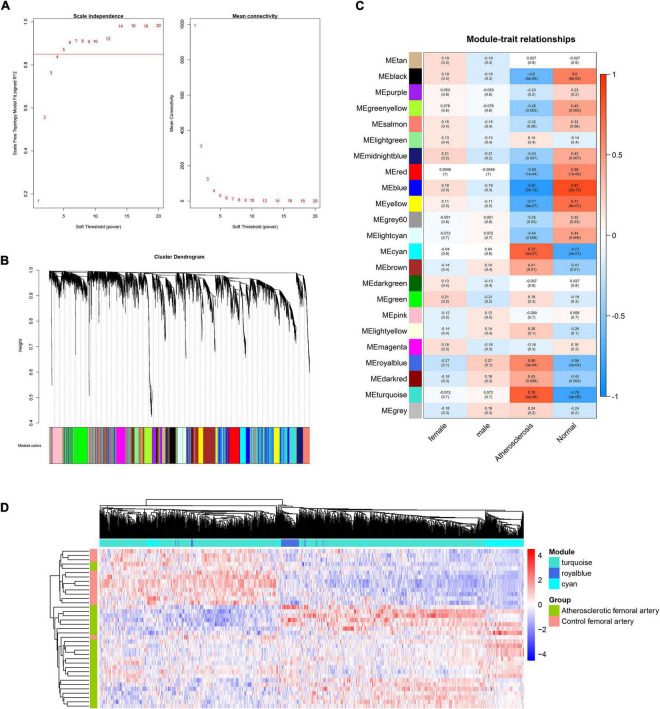
WGCNA analysis of lncRNAs in femoral atherosclerosis. **(A)** The soft threshold power was screened for the lncRNAs matrix. **(B)** The cluster dendrogram of lncRNAs modules. **(C)** The correlation heatmap displays the association between lncRNAs modules and clinical traits. **(D)** Heatmap exhibits the expression of lncRNAs in screened modules.

To construct the scale-free network of lncRNAs, β = 5 was chosen and 23 gene modules were identified (Figures 3A,B). The cyan, royal blue, and turquoise modules were identified as key modules based on the threshold criteria of *r* > 0.5, *p* < 0.05 (Figure 3C). The majority of module lncRNAs indicated a differential expression trend in the two groups (Figure 3D).

### Functional Annotation and Enrichment of Module Genes

**FIGURE 4:**
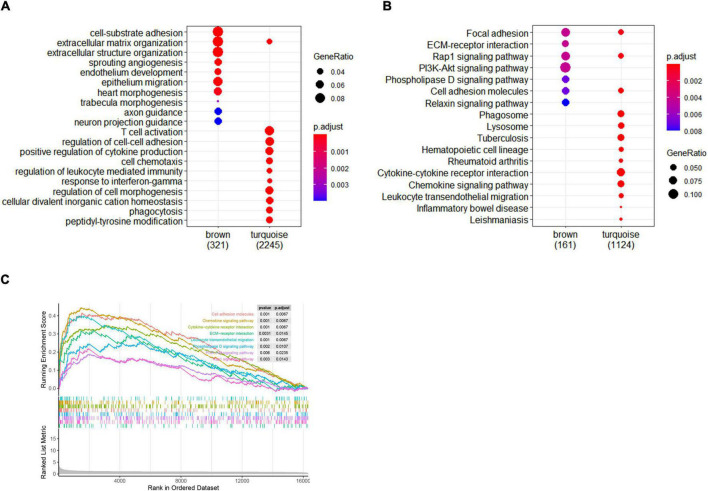
Enrichment analysis of gene modules and GSEA analysis. **(A)** The top 10 GO enrichment analyses of two modules. **(B)** The top 10 signal pathways of KEGG pathway enrichment. Criteria threshold of *p* < 0.05 and *adjusted. p* < 0.05. **(C)** Gene set enrichment analysis in femoral atherosclerosis. Criteria threshold of *adjusted. p* < 0.05.

To further investigate the potential biological function of module genes, GO enrichment and KEGG pathway analysis were performed by the “ClusterProfiler” package. The top 10 results of each module were displayed in the enrichment scatter plots. Regarding biological processes, the genes in the brown module were mainly related to cell-substrate adhesion, extracellular matrix, and structural organization, while the turquoise module mainly focused on immune cell activation (Figure 4A). For KEGG pathway enrichment, focal adhesion, ECM-receptor interaction, *Rap1* signaling pathway, *PI3K-Akt* signaling pathway, *Phospholipase D* signaling pathway, and cell adhesion molecules were remarkable in the brown module. For the genes in the turquoise module, cytokine–cytokine receptor interaction and chemokine signaling pathway were primarily involved (Figure 4B).

### Gene Set Enrichment Analysis

For the purpose of gaining insight into the distinct pathways in femoral atherosclerosis, GSEA was also performed. Pathways were significantly related to cell adhesion molecules, ECM–receptor interaction, chemokine signaling pathway, cytokine–cytokine receptor interaction, leukocyte transendothelial migration, phospholipase D signaling pathway, *PI3K-Akt* signaling pathway, and *Rap1* signaling pathway (Figure 4C). The same pathway enrichment results increase the confidence of modular genes that are principally involved in femoral atherosclerosis.

### Identification of Differentially Expressed Genes and Long Non-coding RNA in Proliferative Vascular Smooth Muscle Cells

**FIGURE 5:**
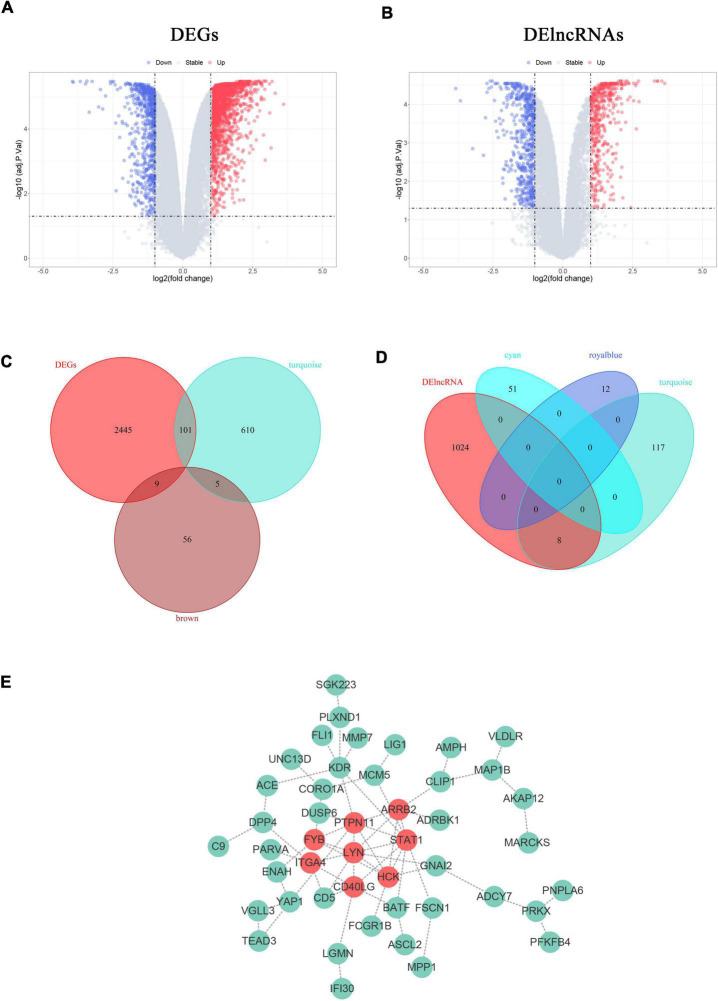
Construction of VSMCs-specific PPI networks. **(A,B)** Volcano plots show the DEGs **(A)** and DElncRNAs **(B)** in proliferative VSMCs. Significantly upregulated genes and lncRNAs are marked in red and those that were downregulated are marked in blue with the threshold of *|log2FC|* ≥ 1 and *adjusted. p* < 0.05. **(B,C)** Venn diagrams display the specific proliferative VSMCs genes **(B)** and lncRNAs **(D)**. **(E)** The PPI network of the defined sp-SMCGs. Each gene is displayed in a green node with the label. The red nodes indicate the common Hub genes of the five algorithms in CytoHubba.

To obtain the expression profiles of genes and lncRNAs in proliferative VSMCs, the selected dataset was re-annotated and analyzed, with 18,087 genes and 12,883 lncRNAs identified. Eventually, 2,555 genes and 1,032 lncRNAs were defined as significant genes according to the threshold criteria of *|logFC|* ≥ 1 and *adjusted*. *p* < 0.05 (Figures 5A,B). Among them, 2,555 genes were identified, with 1,890 upregulated and 665 downregulated genes. For lncRNAs, 459 lncRNAs were upregulated and 573 were significantly downregulated.

### Construction of the Specific Protein-Protein Interaction Network of Proliferative Vascular Smooth Muscle Cells in Femoral Atherosclerosis

Since the functional enrichment revealed that the module genes primarily affect the cellular matrix organization and immune response that might probably induce the phenotypic transition of VSMCs, it was crucial to unravel the specific regulatory network of proliferative VSMCs. To further reveal the potential targets that remain unknown, the key module genes or lncRNAs were considered as the genes or lncRNAs with the threshold of |MM| > 0.8 and |GS| > 0.2. Furthermore, only 716 genes in the turquoise module, 70 genes in the brown module, 51 lncRNAs in the cyan module, 12 lncRNAs in the royal blue module, and 125 lncRNAs in the turquoise module were left. The sp-SMGs and sp-SMlncRNAs were defined as the intersecting genes between the DEGs/DElncRNAs and the key module genes/lncRNAs. The 120 sp-SMGs and 8 sp-SMlncRNAs were eventually identified (Figures 5C,D). The PPI network was established and visualized by utilizing the STRING database and Cytoscape software, which is comprised of 46 nodes and 64 edges (Figure 5E). Eight common hub genes (*LYN, PTPN11, HCK, STAT1, ARRB2, CD40LG, ITGA4*, and *FYB*) were calculated by utilizing five algorithms in the plug-in “Cytohubba” (Supplementary Table 1).

### Construction of the Specific Competitive RNA and RNA Binding Proteins Network of Proliferative Vascular Smooth Muscle Cells in Femoral Atherosclerosis

**FIGURE 6:**
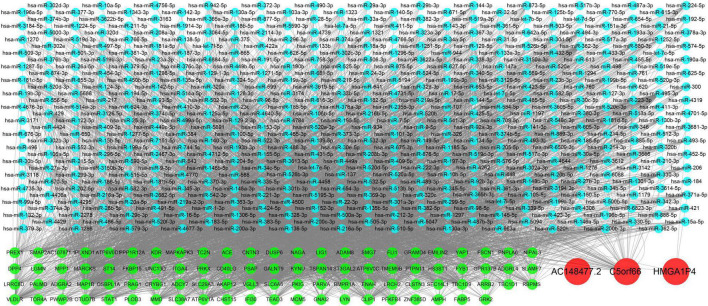
Construction of VSMCs specific ceRNA network. Green nodes indicate the mRNAs, blue nodes indicate the predicted miRNAs, and the red nodes indicate the lncRNAs.

**FIGURE 7:**
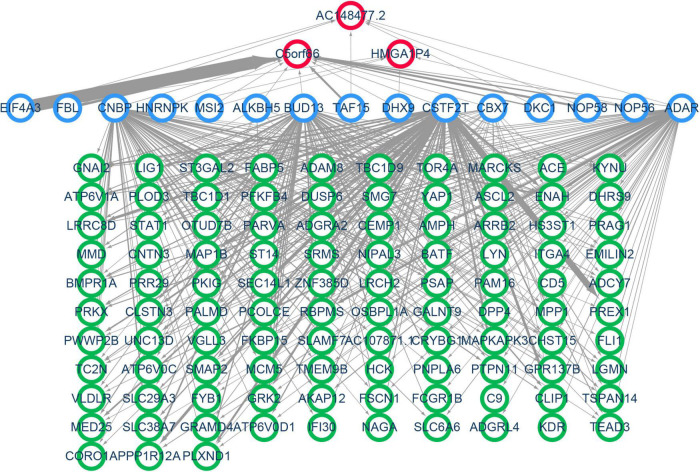
Construction of VSMC-specific RBP network. Nodes with a green border represent the mRNAs, nodes with a blue border indicate the predicted RNA binding proteins, and red border nodes reveal the lncRNAs. The thickness of the edge reflects the number of binding sites.

**FIGURE 8:**
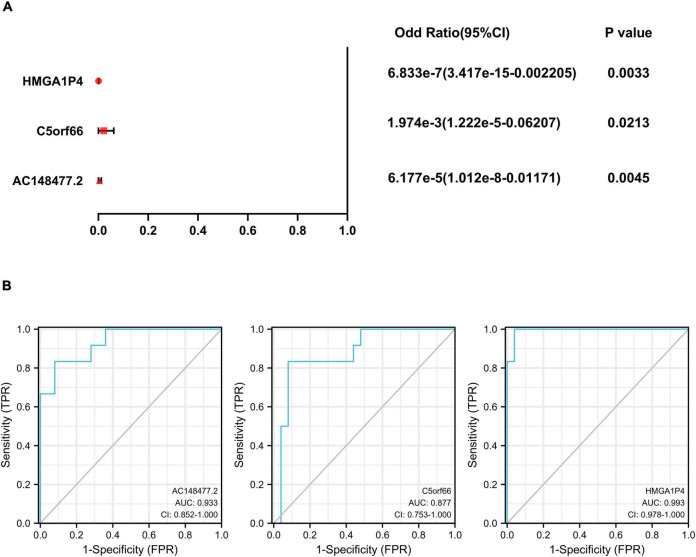
Univariate logistic regression and ROC curves of 3 lncRNAs. **(A)** Univariate logistic regression analysis of *HMGA1P4*, *C5orf66*, and *AC148477.2*. **(B)** AUC of ROC curves of *HMGA1P4*, *C5orf66*, and *AC148477.2*.

A specific ceRNA network was constructed to explore the function of the lncRNAs-miRNAs-mRNAs regulatory axis in proliferative VSMCs, which contributed to the exacerbation of femoral atherosclerosis. Screened from the database, 78 pairs of miRNA-lncRNA interactions and 3,654 pairs of miRNAs-mRNAs interactions were found. The specific ceRNA network, which comprised 535 nodes and 3,731 edges, was constructed and visualized by the Cytoscape software (Figure 6). Three lncRNAs (*HMGA1P4*, *C5orf66*, and *AC148477.2*) were identified in the network. The expression pattern between lncRNAs and target genes was shown in the correlation heatmap (Supplementary Figure 2). To further explore the direct binding of lncRNAs, RBP-lncRNA pairs were retrieved from ENCORI. A RBP network that contained 121 nodes and 382 edges was established (Figure 7). Next, univariate logistic regression analysis was performed, which showed that all three lncRNAs were favorable factors in femoral atherosclerosis patients (Figure 8A, *HMGA1P4*, OR = 6.833e^–7^, 95% CI: 3.417e^–15^–0.002205, *p* = 0.0213; *C5orf66*, OR = 1.974e^–3^, 95% CI:1.222e^–5^–0.06207, *p* = 0.0213; *AC148477.2*, OR = 6.177e^–5^, 95% CI: 1.012e^–8^–0.01171, *p* = 0.0045). The AUC of ROC curves for the three lncRNAs were 0.933, 0.877, and 0.993 (Figure 8B).

### Validation of Three Long Non-coding RNA in Arteriosclerosis Obliterans Arteries and Proliferative Vascular Smooth Muscle Cells

**FIGURE 9:**
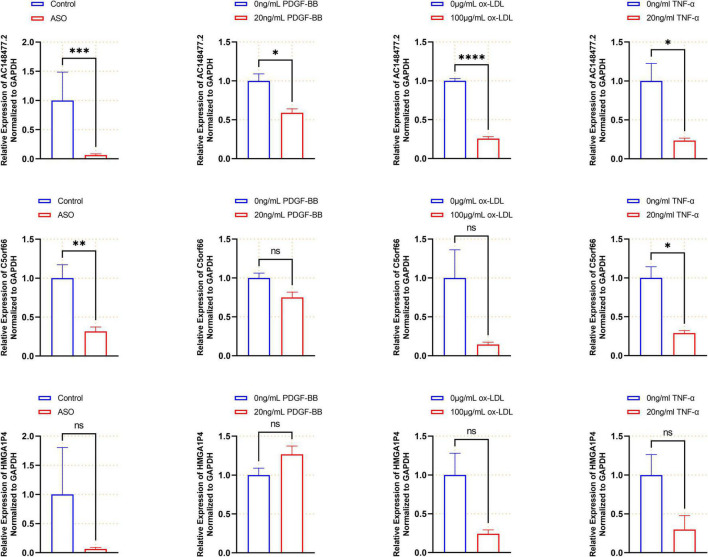
Validated expression of the three lncRNAs. Validation of each lncRNAs in the artery samples (*n* = 12 in each group), PDGF-bb (*n* = 3), ox-LDL (*n* = 4), and TNF-α (*n* = 4) treated VSMCs are shown by histogram (*p < 0.05, **p < 0.01, ***p < 0.001, ****p < 0.0001).

The expression of these lncRNAs was subsequently validated in the artery samples (Figure 9). All three lncRNAs were down-regulated in the ASO group. The expression levels of *AC14847.6* and *C5orf66* were significantly lower in the atherosclerotic arteries. To further confirm the expression of the three lncRNAs in the proliferative VSMCs, three *in vitro* models were set up. When treated with 20 ng/ml PDGF-BB, the expression of *AC14847.2* was significantly lower than that without PDGF-BB treated, which was consistent with the results obtained from the tissues. In response to the 100 μg/ml ox-LDL stimulation, the expression of all lncRNAs decreased, whereas the expression of *AC148477.2* was significantly decreased. To simulate inflammation that induces VSMCs proliferation, TNF-α with a concentration of 20 ng/ml was used. Both *AC148477.2* and *C5orf66* tended to have a relatively lower expression in TNF-α treated VSMCs than in normal VSMCs. These results indicated that only *AC148477.2* might play a role in all stages of proliferation in VSMCs.

### Association of Three Long Non-coding RNA With Immune Infiltration Landscape in Femoral Atherosclerosis

**FIGURE 10:**
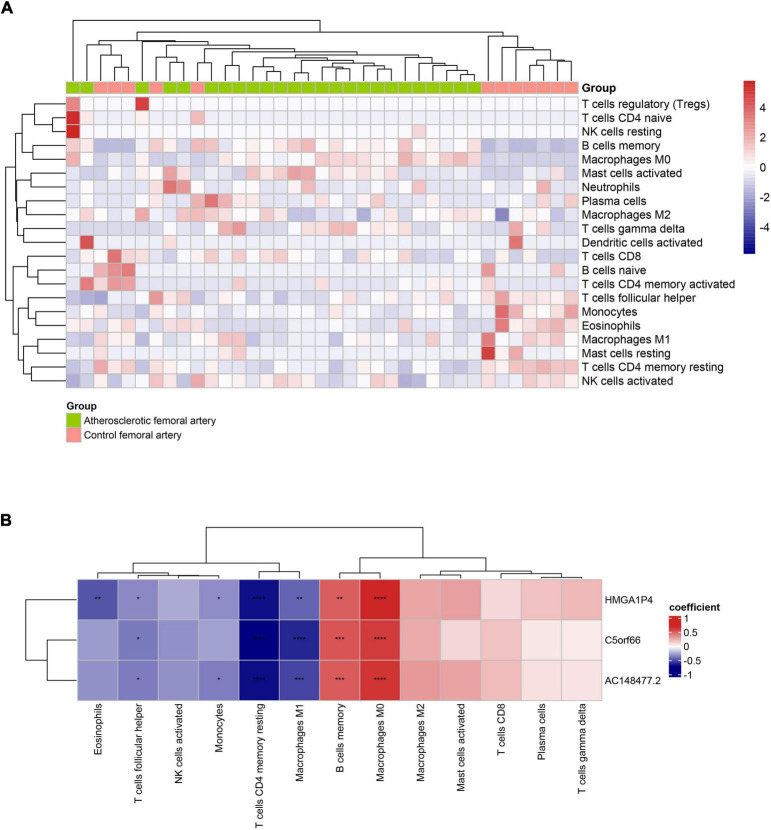
Immune infiltration of 37 artery samples based on CIBESORT analysis tool. **(A)** The infiltrated immune landscape is visualized in the heat map. **(B)** The correlation heat map presents the association between lncRNAs and immune cells. The red square represents positive correlation and the blue square represents negative correlation (*p < 0.05, **p < 0.01, ***p < 0.001, ****p < 0.0001).

According to the assessment of ssGSEA scores, multitudinous immune cell types had a relatively higher infiltration in the femoral atherosclerotic arteries (Supplemental Figure 3A). Macrophages, which contribute to the progression of atherosclerosis, showed higher infiltration based on algorithms. To classify the infiltration of different subtypes of macrophages, analysis was conducted under the CIBERORT website tool. Remarkably, the M0 and M2 macrophages were inclined to show lower infiltration in most of the femoral atherosclerosis samples than in normal femoral arteries, which was opposite to the distribution of the M1 subtype of macrophages (Figure 10A). A relationship between the three lncRNAs and immune cells based on Pearson’s correlation analysis was subsequently conducted. These lncRNAs were positively correlated to the great mass of immune cells, including macrophages, CD4+ T cells, CD8+ T cells, B cells, and natural killer cells (Supplemental Figure 3B). When divided into three types of macrophages, Pearson’s correlation analysis revealed that the three lncRNAs were positively correlated with M0 and M2 macrophages and negatively correlated with M1 macrophages (Figure 10B). To further investigate the expression pattern of these lncRNAs in macrophage polarization, public datasets were analyzed and visualized in histograms (Supplementary Figure 4). Although *C5orf66* was markedly decreased in M1 macrophages compared with M0 macrophages of the GSE162698 dataset, more results indicated that these three lncRNAs did not vary significantly from different types of macrophages.

## Discussion

Atherosclerosis is a multi-step disease that has varying risk factors, including accumulation of macrophages, pro-inflammatory cytokines, endothelial dysfunction, and proliferation of VSMCs (29). In response to the stimulation of the inflammatory microenvironment, activated VSMCs have an enhanced proliferative ability, which leads to the formation of neointima and promotes atherosclerotic lesion progression. Extensive proliferation of VSMCs can produce and secrete extracellular matrix that triggers the thickening of blood vessel walls and narrows the vascular lumen. In response to ox-LDL and activated macrophages, VSMCs can migrate to the arterial intima and produce fibrous tissue. Moreover, these activated VSMCs can also produce pro-inflammatory cytokines and phagocytize lipoproteins (30). According to recent studies, lncRNAs play a crucial role in regulating atherosclerosis (31, 32).

Previous studies have elucidated the role of lncRNAs in atherosclerosis, but few have focused on the lncRNA-related ceRNA network, especially in the extensive proliferative VSMCs. With the aim of distinguishing the pathogenic genes of atherosclerosis, the DEGs obtained by setting the screening criteria are commonly used. But some inherent disadvantages exist. Based on the artificial screening thresholds, masses of biologically functional genes without significant changes in their expression levels may be excluded. In addition, genes with significantly altered expression levels are not necessarily driving genes. In this study, the trait-related genes as well as lncRNAs that were identified by WGCNA are well established. The two modules that were identified were significantly related to femoral atherosclerosis. The brown module was mainly involved in an extracellular matrix formation, and the turquoise module was enriched in cytokine production and inflammatory response. Interestingly, a majority of module genes were also differentially expressed between the two groups. Activated VSMCs can also produce pro-inflammatory cytokines and form the extracellular matrix that contributed to the development of femoral atherosclerosis (33, 34). In addition, the *PI3K-Akt* and *Rap1* signaling pathways were both significantly enriched based on GSEA analysis, which implied the increased proliferative activity of VSMCs. Phosphatidylinositol 3 kinase (*PI3K*) is a key molecule in the initiation of signal transduction pathways after the binding of extracellular signals to cell surface receptors. A growing number of studies have indicated that the *PI3K-Akt* signaling pathway plays a crucial role in the pathophysiological process of atherosclerosis (35). Suwanabol et al. have reported that TGF-β stimulates VSMCs proliferation through activating p38 and Akt in the presence of elevated levels of *Smad3* (36). *Rap1* (Ras-associated protein 1), which is a small GTPase that belongs to the Ras family of GTPases, is related to many of the hallmarks of cancer (37). Perdomo et al. have reported that *Rap1* was overexpressed in the large extracellular vesicles (EVs) of atherosclerotic patients (38). These EVs significantly promoted the migration and proliferation of VSMCs. Previous studies have demonstrated that *Rap1* protein levels are upregulated in PDGF-bb treated VSMCs (39). Recently, a specific binding protein of *Rap1*, named *Epac1*, was reported to facilitate the migration of VSMCs (40). The deficiency of *Epac1* significantly attenuated the neointima after femoral artery injury in mice. The results above demonstrate that several genes were changed and participated in the phenotype plasticity.

To further verify the specific lncRNAs and genes of phenotype transition of VSMCs, which are concealed in the gene modules, the dataset of proliferative VSMCs was employed. Therefore, we initially structured the lncRNA-mediated ceRNA network to seek the potential diagnosis target, which focused on the pathological proliferation of VSMCs within femoral atherosclerosis. Three lncRNAs, including *HMGA1P4*, *C5orf66*, and *AC148477.2* were ultimately identified in the specific ceRNA network, as well as the specific RBP network. A limited number of studies revealed that *HMGA1P4* was functional in gastric cancer (41, 42). The *HMGA1P4* levels were up-regulated in DDP-resistant GC tissues and cells that might trigger the progression of DDP-resistance by upregulating MDR-related genes and downregulating apoptosis-related genes (43). However, no existing evidence reports the function of *HMGA1P4* in VSMCs. *HMGA1P4* was found to be down-regulated in ASO arteries, ox-LDL, and TNF-treated VSMCs in this study, but no significance was found. Unfortunately, the function of *C5orf66* has not been reported despite having a significantly lower expression in atherosclerotic arteries and proliferative VSMCs. Interestingly, *AC148477.2* was concomitantly down-regulated in both tissues and cells, which suggested that *AC148477.2* might be involved in the whole process of phenotype switch in VSMCs. As a potential therapeutic target, the overexpression of *AC148477.2* may inhibit the excessive proliferation of VSMCs in ASO. In the RBP network, the interaction between lncRNAs suggests that mutual regulation might exist in the process of atherosclerosis. After verification, we may have identified lncRNAs that regulate different stages of VSMCs in atherosclerosis, which can be considered regulatory targets for further research. More experimental evidence is still needed to reveal the regulatory mechanism of these functional lncRNAs in VSMCs.

Macrophages, which have developed remarkable plasticity in switching inflammatory responses, play a crucial part in all stages of atherosclerosis (8). Stimulated by multiple factors in the microscopic environments, with atherosclerotic plaque, macrophages transform into different phenotypes with differential expression patterns (44). The classically activated macrophages (M1) that could be induced by ligand lipopolysaccharide (LPS) *in vitro*, are characterized as the pro-inflammatory phenotype. The other activated macrophages (M2), which could be induced by IL-4 and IL-13, are strongly related to the anti-inflammatory phenotype. Emerging evidence shows that different subsets of macrophages are located in various lesions of the atherosclerotic plaque. Both the makers of M1 and M2 were observed in the fibrous cap (45). Higher levels of M1 macrophages could be detected in the vulnerable plaque, which suggests that a high level of M1 macrophage infiltration or high M1/M2 ratio is related to a high risk of plaque rupture (45–48). However, studies supported that the M2 macrophage was more likely located in the stable plaque region and next to the calcified areas rather than the lipid core (49, 50). The M2 macrophages were found to functionally prevent the progression of atherogenesis by reducing the plaque size, enhancing the plaque stability, and promoting the VSMCs to maintain the contractile state (51). In the current study, we evaluated the immune infiltration of the 37 artery samples through ssGSEA and the CIBERSORT method. Results of ssGSEA indicated that a relatively higher distribution of a large proportion of immune cells, including macrophages, existed in the atherosclerotic femoral arteries. It is surprising that M0 and M2 macrophages showed a higher infiltration in femoral atherosclerosis while M1 macrophages showed a relatively lower infiltration. This may be explained by the different properties of the plaque. Although peripheral arterial occlusive disease shares most of the risk factors with coronary artery disease, the pathogenesis of atherosclerotic disease in specific locations is different. A histological study of the common femoral and SFA plaque from the Dutch Athero-Express Biobank revealed fibrotic plaque with collagen, SMs, and calcification (52). The predominant cells in atherosclerotic lesions of SFA are VSMCs, accompanied by a small number of macrophages. This suggested that the lesions in atherosclerotic artery samples were principally composed of stable plaques. A negative correlation was found between the three lncRNAs and the M1 macrophages, while positive associations were computed among the three lncRNAs and M0 macrophages subtype. Thus, it was intriguing to investigate whether those three lncRNAs, especially *AC148477.2*, might be involved in inhibiting the pro-inflammatory phenotypic transformation of macrophages. Nevertheless, due to the small sample size of the dataset, the results of Pearson’s correlation analysis may show bias. Further experimental evidence is needed to explain whether these lncRNAs could regulate the activation of macrophages.

Thus, we identified three specific lncRNAs and constructed the ceRNA and RBP networks in femoral atherosclerosis, which revealed the potential molecular mechanism of proliferative VSMCs. After validation in artery samples and *in vitro* models, we found that *AC148477.2* might be regarded as a novel therapeutic target due to its ability to regulate the intimal hyperplasia that is induced by excessive proliferation of VSMCs. Nevertheless, some innate limitations still exist in our study. With the increasing popularity of endovascular therapy, the number of patients requiring amputation is decreasing. It is very challenging to collect amputation samples, but a larger sample size would be more helpful to support the results of the bioinformatics mining. Further experimental verification should be conducted to determine the exact molecular mechanisms of the alternative lncRNAs.

In conclusion, this study systematically demonstrated the regulatory network which mainly focused on the proliferative VSMCs within femoral atherosclerosis. This innovative research might help in unraveling the pivotal genes that might serve as independent biomarkers of femoral atherosclerosis.

## Data Availability Statement

The original contributions presented in this study are included in the article/Supplementary Material, further inquiries can be directed to the corresponding author.

## Ethics Statement

The studies involving human participants were reviewed and approved by the IEC for Clinical Research and Animal Trials of The First Affiliated Hospital of Sun Yat-sen University. The patients/participants provided their written informed consent to participate in this study.

## Author Contributions

CY and SW contributed to the concept of the study. KW and YY performed the bioinformatics analysis and jointly make effort in the manuscript preparation. LH assisted in the experiment. RW and RH participated in the manuscript review. All authors contributed to the article and approved the submitted version.

## Conflict of Interest

The authors declare that the research was conducted in the absence of any commercial or financial relationships that could be construed as a potential conflict of interest.

## Publisher’s Note

All claims expressed in this article are solely those of the authors and do not necessarily represent those of their affiliated organizations, or those of the publisher, the editors and the reviewers. Any product that may be evaluated in this article, or claim that may be made by its manufacturer, is not guaranteed or endorsed by the publisher.

## Footnotes

1
http://www.ncbi.nlm.nih.gov/geo

## Abbreviations

ASO: Arteriosclerosis obliterans
SFA: Superficial femoral artery
VSMCs: Vascular smooth muscle cells
lncRNAs: Long non-coding RNAs
ceRNA: Endogenous competitive RNA
WGCNA: Weighted gene correlation network analysis
DEGs: Differentially expressed genes
DElncRNAs: Differentially expressed lncRNAs
TOM: Topological overlap matrix
ME: Module eigengene
MM: Module membership
GS: Gene significance
GO: Gene Ontology
KEGG: Kyoto Encyclopedia of Genes and Genomes
GSEA: Gene set enrichment analysis
PPI: Protein-protein interaction
HASMCs: Human aortic smooth muscle cells
ssGSEA: Single-sample gene set enrichment analysis
sp-SMGs: Specific VSMCs module genes
sp-SMlncRNAs: specific VSMCs module lncRNAs
GEO: gene expression omnibus
RBP: RNA binding proteins.
