# Supplementary material for: The Long Non-coding RNA AC148477.2 Is a Novel Therapeutic Target Associated With Vascular Smooth Muscle Cells Proliferation of Femoral Atherosclerosis
Source: Front Cardiovasc Med. 2022 Jul 6;9:954283. doi: 10.3389/fcvm.2022.954283 (PMC9297286; doi:10.3389/fcvm.2022.954283)
Supplement: Supplementary file 1 [file Table_1.DOCX]

Supplementary Material

# Supplementary Table

Table 1

The top 10 Hub genes in the PPI network

| **Category** | **Rank methods in CytoHubba** | | | | |
| --- | --- | --- | --- | --- | --- |
|  | **MCC** | **MNC** | **EPC** | **Degree** | **Closeness** |
| 1 | ***LYN*** | ***LYN*** | ***LYN*** | ***LYN*** | ***STAT1*** |
| 2 | ***PTPN11*** | ***STAT1*** | ***STAT1*** | ***STAT1*** | ***LYN*** |
| 3 | ***HCK*** | ***HCK*** | ***PTPN11*** | ***PTPN11*** | ***PTPN11*** |
| 4 | ***STAT1*** | ***PTPN11*** | ***HCK*** | ***HCK*** | ***HCK*** |
| 5 | ***ARRB2*** | ***CD40LG*** | ***ARRB2*** | ***ARRB2*** | ***ARRB2*** |
| 6 | ***CD40LG*** | ***ARRB2*** | ***CD40LG*** | ***CD40LG*** | ***CD40LG*** |
| 7 | ***ITGA4*** | ***ITGA4*** | ***FYB*** | *KDR* | *KDR* |
| 8 | ***FYB*** | *CD5* | ***ITGA4*** | ***ITGA4*** | ***FYB*** |
| 9 | *KDR* | ***FYB*** | *KDR* | ***FYB*** | ***ITGA4*** |
| 10 | *CD5* | *TEAD3* | *CD5* | *YAP1* | *BATF* |

Table 2

The primers of all genes.

| Gene | Forward primer | Reverse primer |
| --- | --- | --- |
| HMGA1P4 | CACACCCTCCTCCACTGTCCTG | CCTCCTGCTTTGTTTCCTGTACCC |
| C5orf66 | AAGATGCCAAACACAGACCCACTC | TGGAATCACAACGCAGCAGACAG |
| AC148477.2 | AAGAGGAAGTGGAGGCTGAGTGG | GATGGAAGAGCACGCAGGGAAG |
| GAPDH | GCACCGTCAAGGCTGAGAAC | TGGTGAAGACGCCAGTGGA |
