# Supplementary material for: The Long Non-coding RNA AC148477.2 Is a Novel Therapeutic Target Associated With Vascular Smooth Muscle Cells Proliferation of Femoral Atherosclerosis
Source: Front Cardiovasc Med. 2022 Jul 6;9:954283. doi: 10.3389/fcvm.2022.954283 (PMC9297286; doi:10.3389/fcvm.2022.954283)
Supplement: Supplementary file 2 [file Table_2.DOCX]

Supplementary Material

# SupplementaryFigures
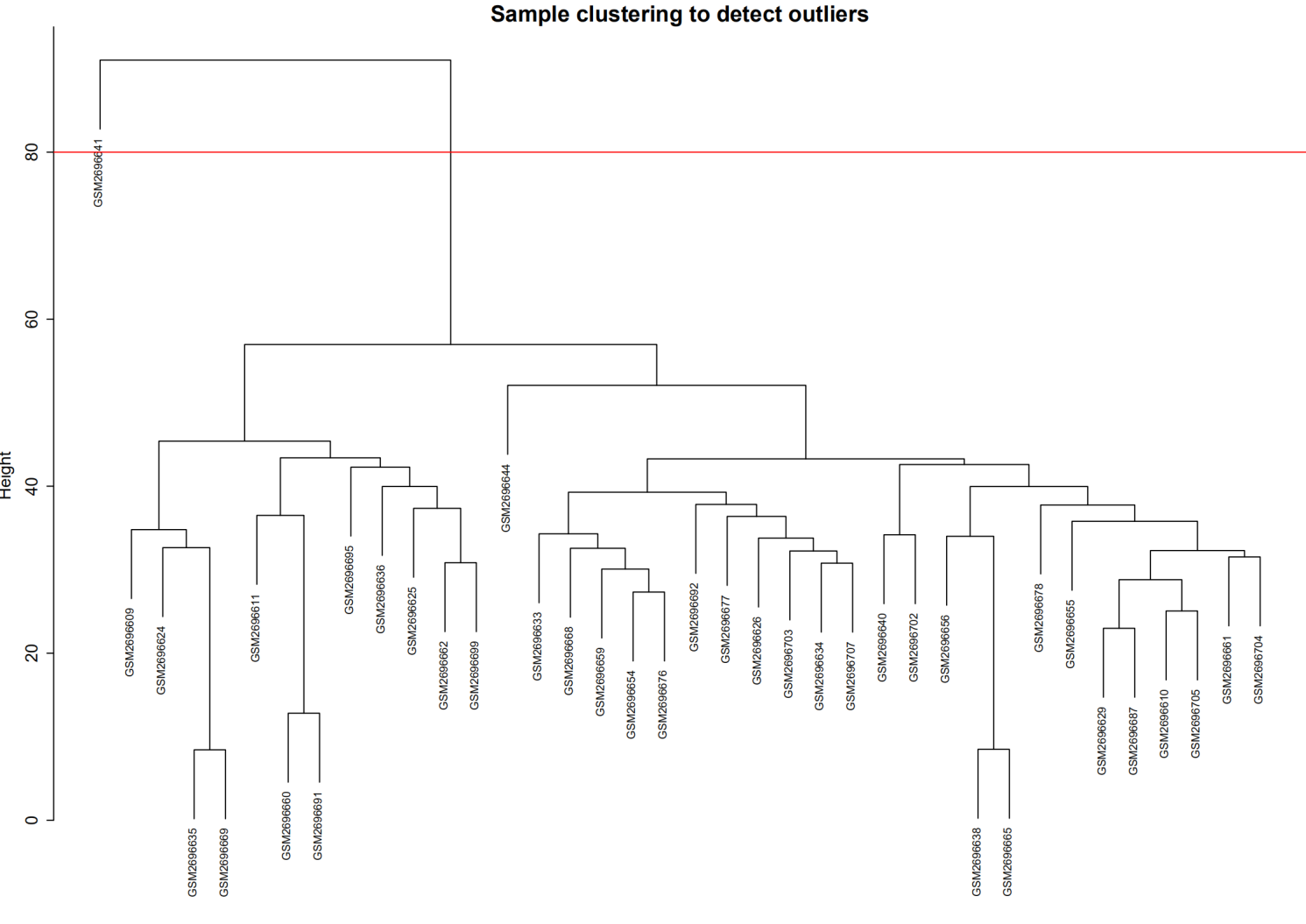
Supplementary Figure 1. A sample clustering tree diagram shown the separate samples. The cut line of Height 80 was chosen with the sample GSM296641 removed.


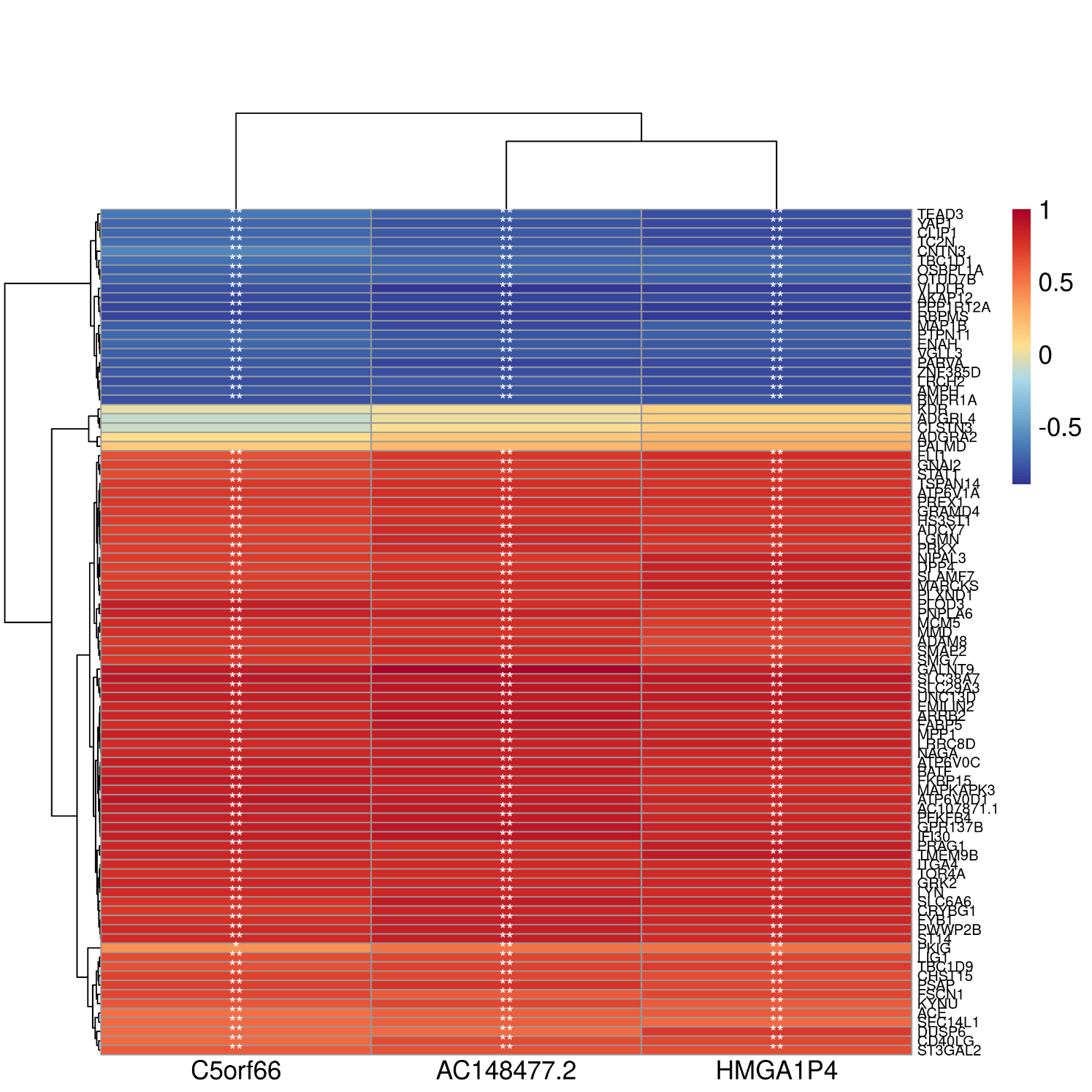
**Supplementary Figure 2.** A correlation heatmap between lncRNAs and mRNA in ceRNA network. Col names indicated the lncRNAs and row names indicated the mRNA. (** *p* < 0.01)


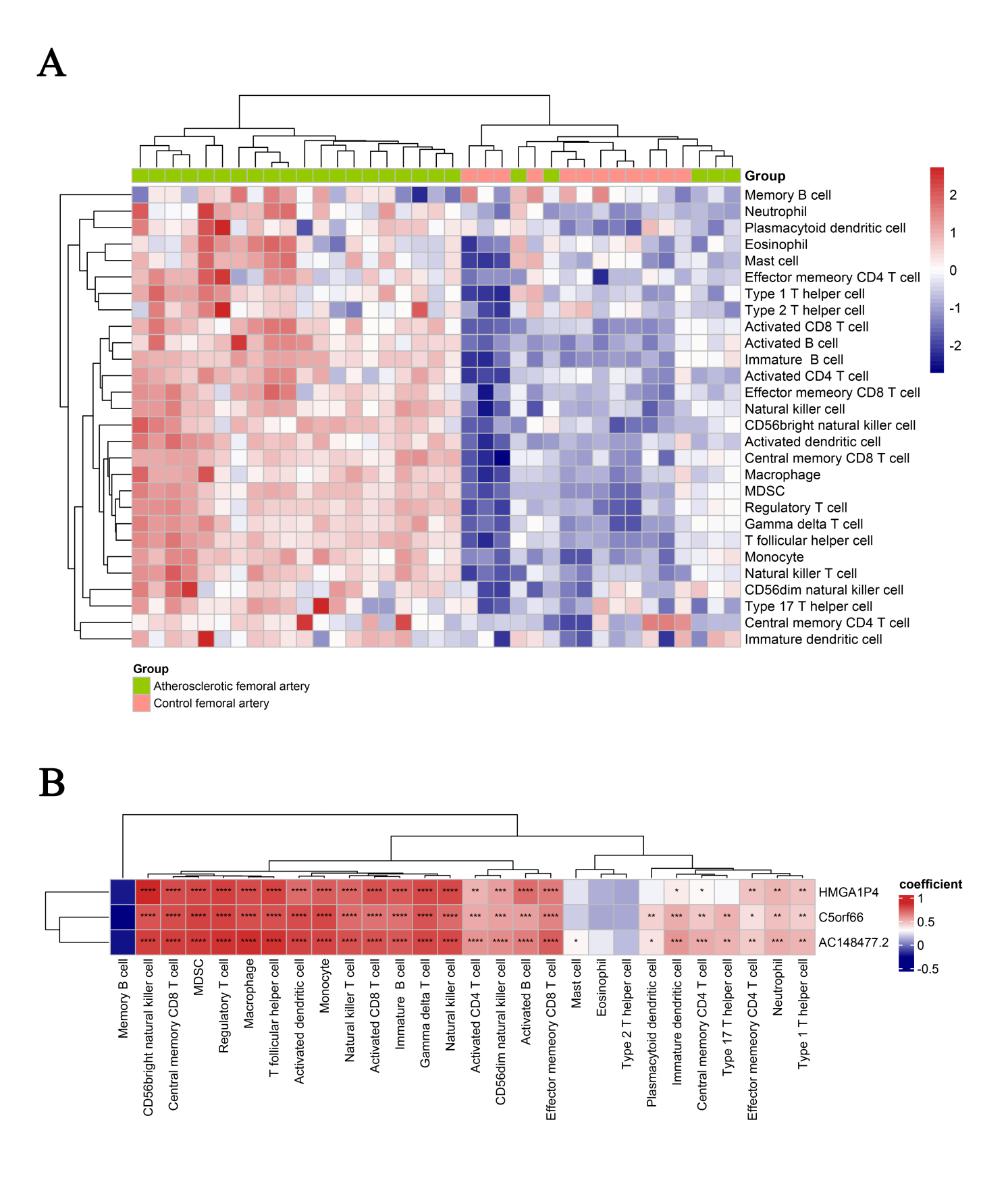


**Supplementary Figure 3.** Immune infiltration of the artery samples analyzed by ssGSEA. (A) The heat map displayed the infiltration of immune cells in the artery samples . (B) Correlation heat map revealed the relationship between lncRNAs and immune cells. (* *p* < 0.05, ** *p* < 0.01, *** *p* < 0.001, **** *p* < 0.0001)


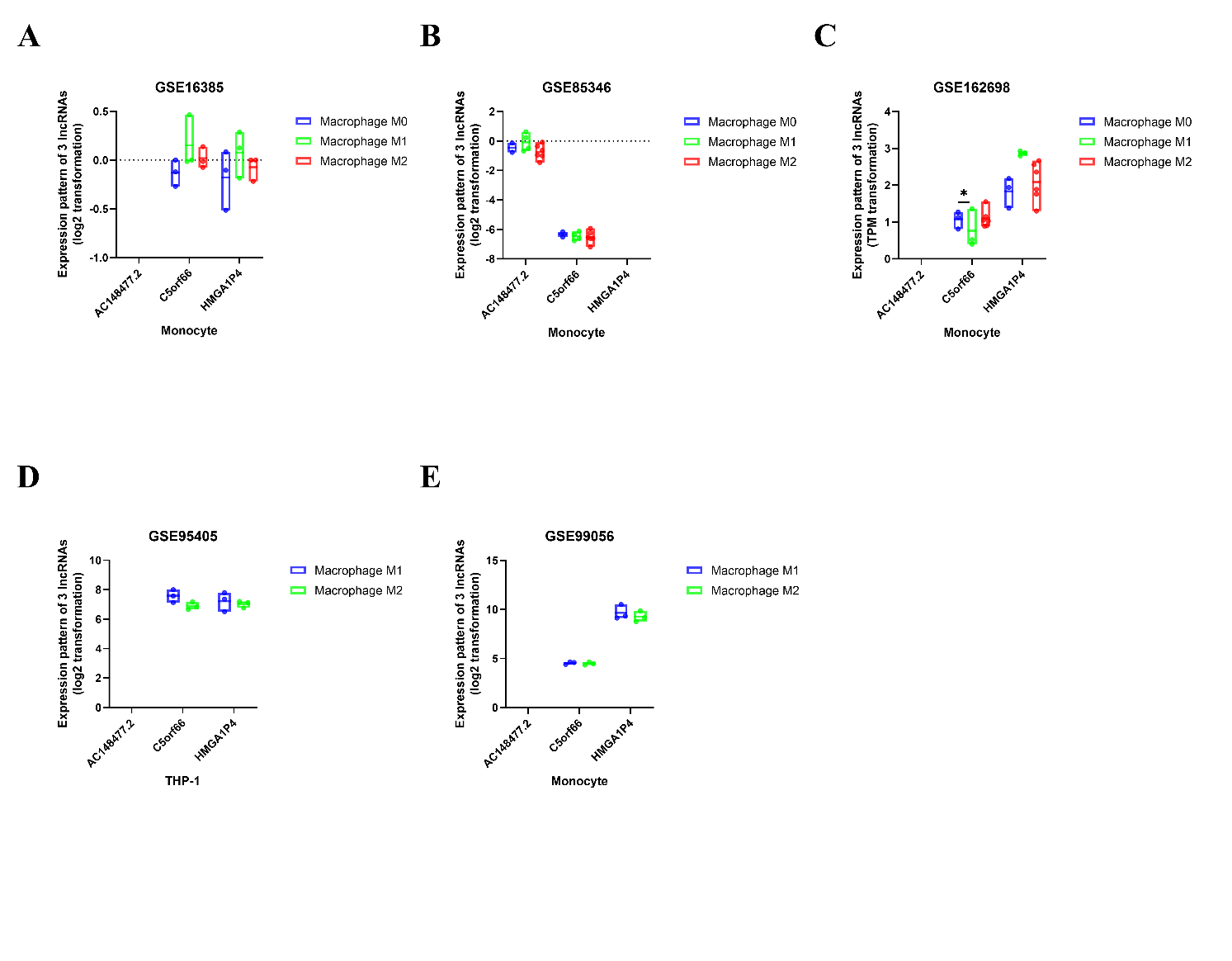


**Supplementary Figure 4.** Expression pattern of 3 lncRNAs in macrophages. (A) PBMCs were treated with IFN-γ (100ng/ml) and TNF-α (50ng/ml) for M1 polarization and IL-4 (100ng/ml) for M2 polarization. (B) PBMCs were treated with LPS (10ng/ml) and IFN-γ (50ng/ml) to induce M1 polarization and IL-4 (10ng/ml), IL-1β (10ng/ml) and IL-10 (10ng/ml) to induce M2 polarization. (C) PBMCs were stimulates with IFN-γ (20ng/ml) and LPS (100ng/ml) for M1 polarization. M2 polarization was induced by cultured with IL-4 (20ng/ml) and IL-10 (20ng/ml). (D) THP-1 differentiated macrophages were supplemented with LPS (10pg/ml) and IFN-γ (20ng/ml) to polarized to M1 macrophages. M2 state macrophages were induced by treating with IL-4 (20ng/ml). (E) PBMCs were treated with GM-CSF to differentiate into M1 macrophages and treated with M-CSF to induce M2 macrophages. (* *p* < 0.05)
